# MRI Reflects Meningioma Biology and Molecular Risk

**DOI:** 10.3390/cancers17223665

**Published:** 2025-11-15

**Authors:** Julian Canisius, Julia Schuler, Maria Goldberg, Olivia Kertels, Marie-Christin Metz, Chiara Negwer, Igor Yakushev, Bernhard Meyer, Stephanie E. Combs, Jan S. Kirschke, Denise Bernhardt, Benedikt Wiestler, Claire Delbridge

**Affiliations:** 1 Department of Diagnostic and Interventional Neuroradiology, Klinikum Rechts der Isar, School of Medicine and Health, Technical University of Munich, 81675 Munich, Germany; olivia.kertels@tum.de (O.K.); marie.metz@tum.de (M.-C.M.); jan.kirschke@tum.de (J.S.K.); 2 Institute of Pathology, TUM School of Medicine and Health, Technical University of Munich, 81675 Munich, Germany; julia.schuler@tum.de; 3 Department of Neurosurgery, Klinikum Rechts der Isar, School of Medicine and Health, University Hospital of TUM, 81675 Munich, Germany; maria.goldberg@tum.de (M.G.); chiara.negwer@tum.de (C.N.); bernhard.meyer@tum.de (B.M.); 4 Department of Nuclear Medicine, School of Medicine, TUM University Hospital, Technical University of Munich, 81675 Munich, Germany; igor.yakushev@tum.de; 5 Department of Radiation Oncology, TUM School of Medicine, TUM University Hospital Rechts der Isar, Technical University of Munich, 81675 Munich, Germany; stephanie.combs@tum.de (S.E.C.); denise.bernhardt@tum.de (D.B.); 6 AI for Image-Guided Diagnosis and Therapy, TUM School of Medicine and Health, Technical University of Munich, 81675 Munich, Germany; b.wiestler@tum.de

**Keywords:** meningioma, magnetic resonance imaging, radiomics, machine learning, radiogenomics, cIMPACT-NOW update 8, DNA methylation, copy number variation, chromosome 1p status, molecular risk stratification, tumor shape analysis

## Abstract

**Background/Objectives:** Large-scale (epi)genomic studies have substantially advanced our understanding of the molecular landscape of meningiomas, most recently embedded in the cIMPACT-NOW update 8. As a result, molecular data are increasingly integrated into risk-adapted treatment algorithms. However, it remains uncertain to what extent non-invasive MRI can capture underlying molecular variation and risk. **Methods:** We assembled a large, single-institution cohort of 225 newly diagnosed meningiomas (WHO grades 1–3) with available preoperative MRI, as well as comprehensive epigenome-wide methylation and copy-number profiling. Tumors were segmented into core and edema regions using a state-of-the-art automated pipeline from the BraTS challenge. Radiomic features were extracted and used to train Random Forest classifiers to predict WHO grade, molecular risk, and specific alterations such as 1p loss in a hold-out test set. **Results:** Our models achieved accuracy above 91% for integrated molecular risk classification, 87.5% for 1p chromosomal status, and 76.8% for WHO grade prediction, with corresponding AUCs of 0.91, 0.90, and 0.89, underscoring the robustness of radiomic features in capturing histopathological and, especially, molecular characteristics. **Conclusions:** Preoperative MRI effectively captures the underlying molecular biology of meningiomas and may enable rapid molecular assessment to inform decision-making and prioritization of confirmatory testing. However, it is not yet ready for clinical use, showing lower accuracy for current WHO grade classification.

## Simple Summary

Meningiomas are the most common primary brain tumors. Molecular testing has become crucial for estimating tumor behavior, but such testing requires tissue and specialized laboratories. This study examines whether information from routine magnetic resonance imaging can indicate key molecular features non-invasively before surgery. Using computer-assisted analysis of tumor shape and texture on preoperative scans, the models differentiated lower from higher molecular risk and identified loss of chromosome 1p with high accuracy, with limited accuracy in distinguishing high-grade meningiomas for the current WHO classification. Therefore, it is not yet ready for clinical use, complementing but not replacing pathology. By offering valuable insights into tumor biology, it may function as an early decision-support tool, supporting counseling and prioritization of confirmatory testing. Prospective studies are needed to validate these results for clinical implementation.

## 1. Introduction

Meningiomas are the most common primary central nervous system (CNS) tumors, representing more than a third of all central nervous system neoplasms [1]. According to the 2021 WHO Classification of Central Nervous System Tumors, most tumors are classified as WHO grade 1 (benign) and generally have a favorable prognosis following complete surgical resection [2,3]. However, WHO grade 2 (atypical) and grade 3 (anaplastic) meningiomas, comprising approximately 15–25% of all cases, are associated with significantly higher recurrence rates and shorter overall survival [4,5,6,7]. Current European Association of Neuro-Oncology (EANO) guidelines recommend surgery as the primary treatment for symptomatic or enlarging meningiomas, with gross total resection as the goal where safely achievable. Subtotal resection is sometimes necessary to preserve neurological function. Adjuvant radiotherapy is recommended for incompletely resected grade 2 meningiomas and for all grade 3 meningiomas, while the benefit after gross total resection of grade 2 tumors remains the subject of ongoing investigation. Nevertheless, clinical outcomes remain heterogeneous even within the same histological grade, underscoring the need for improved risk stratification [3,8]. The variability in prognosis suggests that traditional histopathological grading alone may be insufficient for optimal risk stratification, highlighting the need for additional biomarkers to guide treatment decisions [8,9].

The landscape of meningioma classification has undergone significant evolution with the introduction of molecular parameters in the 2021 WHO Classification of Central Nervous System Tumors (CNS5) [2]. Most recently, the cIMPACT-NOW update 8 has provided crucial clarifications on molecular risk parameters and grading recommendations, beyond the scope of the 2021 WHO classification [10]. This update specifically emphasizes the clinical significance of chromosomal arm 1p deletion in combination with 22q deletion and/or NF2 oncogenic variants, proposing CNS WHO grade 2 assignment for cases with CNS WHO grade 1 morphology harbouring these molecular alterations. Additionally, this update provides guidance on CDKN2A/B homozygous deletions, TERT promoter mutations, and clarifies that oncogenic variants in TRAF7, AKT1, KLF4, SMO, and POLR2A genes, while prognostically favorable, should not be used as strict grading criteria but rather to inform clinical decision-making.

The integration of molecular parameters into meningioma classification presents both opportunities and challenges for clinical practice [11]. While these advances enable more precise risk stratification and potentially personalized treatment approaches, they require invasive tissue sampling and sophisticated molecular testing infrastructure. Non-invasive prediction of molecular characteristics using advanced imaging—particularly MRI radiomics—could bridge this gap by providing clinically relevant information preoperatively and facilitating risk-adapted treatment planning [12,13].

Radiomics, the high-throughput extraction of quantitative features from medical images, has emerged as a promising approach for non-invasive tumor characterization [14,15]. If validated, such approaches could enable risk-adapted preoperative counseling and treatment planning without the need for molecular testing, particularly in clinical settings lacking routine access to advanced sequencing modalities. Our previous work demonstrated that machine learning algorithms can successfully predict integrated risk scores in WHO grade 2/3 meningiomas using MRI-based radiomic features [16]. Building upon these findings, we evaluated whether MRI-based radiomics can predict molecular risk features and integrated grading criteria newly emphasized in cIMPACT-NOW update 8 [10].

The primary objective of this study was to develop and validate machine learning models for predicting (1) integrated risk classification (high/low), (2) 1p chromosomal status (intact/loss), and (3) WHO grade (1/2/3) using preoperative MRI radiomic features in a large, well-characterized meningioma cohort classified according to cIMPACT-NOW update 8 criteria [10].

## 2. Materials and Methods

### 2.1. Study Population and Ethics

This retrospective study included 225 patients with histopathologically confirmed meningioma who underwent surgical resection at TUM University Hospital between 2018 and 2023. All patients had preoperative MRI examinations and comprehensive molecular characterization according to current standards. The study was conducted in accordance with the Declaration of Helsinki and approved by the institutional review board. Informed consent was waived due to the retrospective design and use of de-identified data, as approved by the local ethics committee.

Inclusion criteria were: (1) histopathologically confirmed meningioma, (2) available preoperative contrast-enhanced T1-weighted and FLAIR MRI sequences, (3) complete molecular characterization including CNV status, and (4) sufficient image quality for radiomics analysis. Patients with prior treatment, incomplete molecular data, or inadequate imaging were excluded.

### 2.2. Histopathological and Molecular Classification

All tumor samples underwent genome-wide DNA methylation analysis using the Illumina EPIC array (Illumina, San Diego, CA, USA). These data were used to determine methylation classes with the Heidelberg classifier (v12.5) (German Cancer Research Center, Heidelberg, Germany) and to infer copy number variations (CNVs), including 1p/22q status and CDKN2A/B homozygous deletions. TERT promoter mutation analysis was performed via Sanger sequencing for higher-grade meningiomas that lacked a CDKN2A/B homozygous deletion. Tumors were classified according to the 2021 WHO criteria, with integrated grading and risk stratification following the recommendations of cIMPACT-NOW update 8. TERT promoter mutation or CDKN2A/B homozygous deletion conferred a CNS WHO grade 3 diagnosis, regardless of histology. All tumors with combined loss of chromosome 1p and 22q were graded as WHO grade 2. The loss of two or more whole chromosomal arms on chromosome 6, 10, 14 and 18 was assigned molecularly high risk. Tumors without these losses and/or isolated loss of 22q were classified as molecularly low risk.

### 2.3. MRI Acquisition and Image Processing

Preoperative MRI examinations were performed on several 1.5T or 3T scanners from major vendors, often also externally (and patients brought images). The following minimum requirements for automated segmentation and features were defined:

• T1-weighted contrast-enhanced sequences (slice thickness ≤ 3 mm);
• FLAIR sequences (slice thickness ≤ 3 mm);
• Additional sequences (T2-weighted, diffusion-weighted) when available.

All images underwent standardized preprocessing, including linear co-registration to a common reference space (SRI24) [17], skullstripping using HD-BET [18], intensity normalization to [0;1] within the brainmask, and automated meningioma segmentation (into necrosis, contrast-enhancing tumor, and edema) using the winning 2023 BraTS Meningioma Segmentation model, implemented in the openly available BrainLesion Suite [19]. For each case, careful manual quality control and, where necessary, manual refinement of the preprocessing and segmentation were performed by an experienced neuroradiologist (J.C.) to ensure accuracy.

### 2.4. Radiomics Feature Extraction

Radiomic features were extracted from both the “tumor core” area of the largest tumor (i.e., contrast-enhancing and cystic/necrotic areas) in both the contrast-enhanced T1 and FLAIR sequences using the pyradiomics package version 3.1.0 [20] and closely following IBSI recommendations [21]. To limit the risk of overfitting, we kept all processing parameters at their recommended defaults in pyradiomics, including the default binwidth for gray value discretization. The feature extraction pipeline included:

1. 3D Shape features: Three-dimensional morphological descriptors including volume, surface area, sphericity, flatness, elongation, and various diameter measurements. In total, these are 16 features, all implemented in the RadiomicsShape() class in pyradiomics.
2. First-order features: Statistical descriptors of voxel intensity distribution including mean, median, standard deviation, skewness, and kurtosis. In total, these are 18 features, calculated through RadiomicsFirstOrder().
3. Second-order texture features: Gray-level co-occurrence matrix (GLCM) derived parameters (23 features), calculated through RadiomicsGLCM().

Feature extraction was performed using validated open-source software with standardized parameter settings to ensure reproducibility. Since first-order and GLCM were calculated both in contrast-enhanced T1 and FLAIR, a total of 98 radiomic features were extracted per patient. A complete feature list is provided in Supplementary Table S1.

### 2.5. Machine Learning Model Development

The dataset was randomly split into training (80%, *n* = 180) and testing (20%, *n* = 45) sets while maintaining class distribution (for WHO grade) balance. This split was kept for all models, and we ensured that no model development or hyperparameter estimation was performed on the test set. We developed a total of three Random Forest-based classification models (for WHO grade, molecular risk, and 1p deletion) using the scikit-learn package. To minimize the risk of overfitting, we left all relevant model hyperparameters (such as the number of trees) at their default values.

### 2.6. Statistical Analysis and Performance Evaluation

Model performance was assessed using standard classification metrics:

• Area Under the Curve (AUC): Primary performance metric;
• Accuracy: Overall classification accuracy;
• F1-score: Weighted harmonic mean of precision and recall;
• Confusion matrices: Detailed error analysis.

Feature importance was analyzed to identify the most predictive radiomics parameters. Statistical significance was set at *p* < 0.05. All analyses were performed using Python 3.10 with scikit-learn, pandas, and matplotlib libraries.

## 3. Results

### 3.1. Cohort Characteristics

The final cohort comprised 225 meningioma patients with a median age of 58 years (range: 25–82 years) and a female predominance (68%, *n* = 153). Tumor locations were distributed as follows: convexity (45%, *n* = 101), skull base (32%, *n* = 72), parasagittal/falcine (15%, *n* = 34), and other locations (8%, *n* = 18). According to cIMPACT-NOW update 8 criteria, the molecular and histological distribution was:

• WHO Grade: Grade 1 (*n* = 156, 69%), Grade 2 (*n* = 57, 25%), Grade 3 (*n* = 12, 6%);
• 1p Status: Intact (*n* = 181, 80%), Loss (*n* = 44, 20%);
• Risk Classification: Low risk (*n* = 185, 82%), High risk (*n* = 40, 18%).

The dataset was randomly split into training (*n* = 180, 80%) and testing (*n* = 45, 20%) sets while maintaining class distribution balance across all three classification tasks.

### 3.2. Model Performance

For binary tasks, a fixed probability threshold of 0.5 was used; for multi-class WHO grade, AUC is reported as macro one-vs-rest.

#### 3.2.1. Risk Classification (High vs. Low)

**Figure 1:**
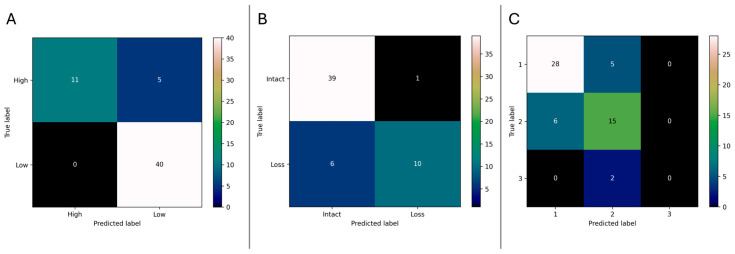
Confusion matrices on the hold-out test set. (**A**) Integrated molecular risk (high vs. low): 0 false positives among low-risk; 5 high-risk predicted as low-risk (sensitivity 68.8%, specificity 100%). (**B**) 1p status (loss vs. intact): 39/40 intact and 10/16 loss correctly classified; 6 false negatives and 1 false positive. (**C**) WHO grade (1/2/3): strong separation for grades 1 and 2; both grade-3 cases (n = 2) predicted as grade 2.

The risk classification model achieved excellent performance with an AUC of 0.91, accuracy of 91.1%, and weighted F1-score of 0.91 (Table 1). The confusion matrix demonstrated particularly strong specificity, with no low-risk cases misclassified as high-risk (0 false positives). Five high-risk cases were incorrectly classified as low-risk, representing a sensitivity of 68.8% and specificity of 100% (Figure 1A). This operating characteristic prioritizes avoidance of overtreatment while maintaining high overall discrimination.

#### 3.2.2. 1p Chromosomal Status Prediction

The 1p status prediction model showed robust performance with an AUC of 0.90, accuracy of 87.5%, and weighted F1-score of 0.87. Analysis of the confusion matrix revealed that the model correctly identified 39 of 40 intact cases and 10 of 16 cases with 1p loss. The primary error pattern was 6 false negatives (1p loss cases predicted as intact) and 1 false positive (Figure 1B).

#### 3.2.3. WHO Grade Classification

The multi-class WHO grade prediction achieved solid performance with a weighted AUC of 0.89, accuracy of 76.8%, and weighted F1-score of 0.75. The confusion matrix showed excellent discrimination between Grade 1 (28/33 correct) and Grade 2 (15/21 correct) tumors. However, both Grade 3 cases (n = 2) were misclassified as Grade 2, likely reflecting the small sample size of Grade 3 tumors in the test set (Figure 1C).

### 3.3. Radiomics Feature Analysis

**Figure 2:**
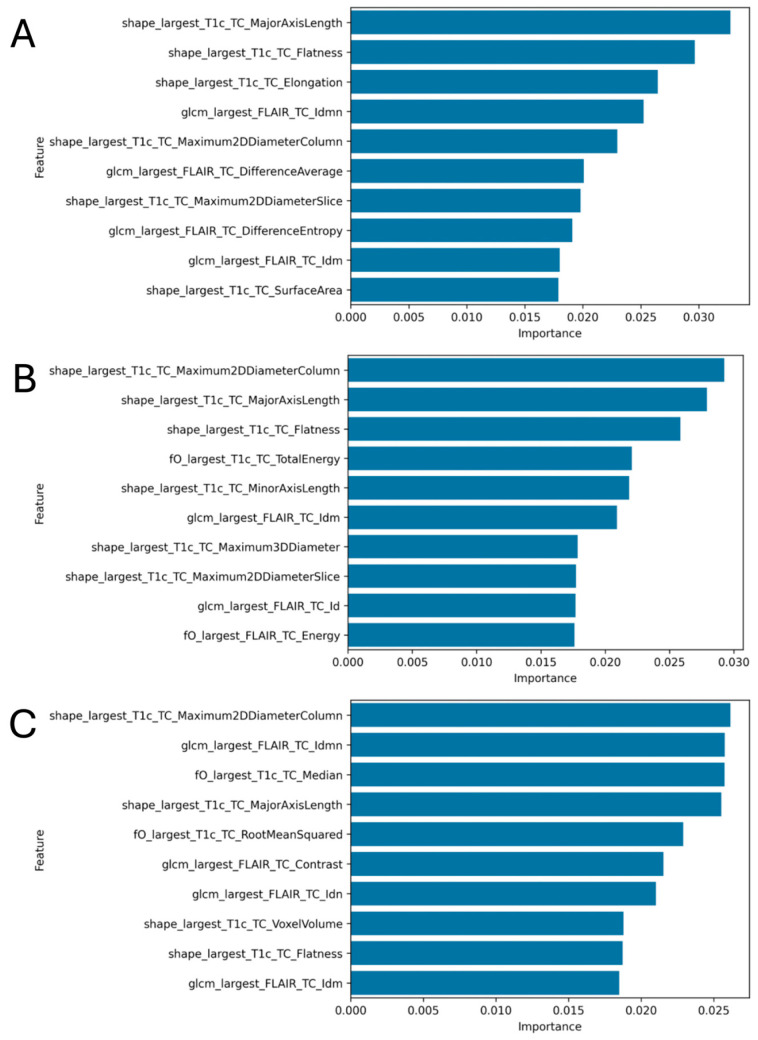
Radiomics feature importance across tasks on the hold-out test set. (**A**) Integrated molecular risk (high vs. low), (**B**) 1p status (loss vs. intact), (**C**) WHO grade (1/2/3). Shape descriptors predominate across models—Flatness, MajorAxisLength, Elongation, and Maximum2DDiameterColumn (prominent in 1p and WHO)—while FLAIR-based GLCM metrics (e.g., Idmn, DifferenceAverage) and first-order measures (e.g., Energy) provide complementary signal. Bars show random-forest feature importances; feature names follow IBSI/pyradiomics conventions.

Across Figure 2A–C, three-dimensional shape features extracted from contrast-enhanced T1 account for most of the model importance, while FLAIR-based GLCM texture and first-order intensity features provide complementary information when morphology alone is less discriminative (Figure 2A–C). In Figure 2A (integrated risk), measures that reflect lesion extent and geometric regularity—MajorAxisLength and Flatness, followed by Elongation—contribute most, with FLAIR-derived Idmn and DifferenceAverage adding complementary information; in this task, Maximum2DDiameterColumn ranks lower within the leading set. In Figure 2B (1p status), the same morphology-led pattern is evident, with Maximum2DDiameterColumn, MajorAxisLength, and Flatness among the top contributors, supplemented by first-order energy from T1c and FLAIR and by FLAIR-GLCM Idm. In Figure 2C (WHO grade), strong shape contributions persist—Maximum2DDiameterColumn, MajorAxisLength, Flatness, and VoxelVolume—while FLAIR-GLCM Idmn and Contrast, together with first-order Median and Root-Mean-Squared, further characterize differences between grades. Overall, morphology—capturing lesion size, extent, and regularity—anchors discrimination across endpoints, and FLAIR-based texture together with first-order intensity features provides complementary discriminative information.

## 4. Discussion

### 4.1. Clinical Significance of Findings

This study demonstrates that MRI-based radiomics can accurately and non-invasively predict molecular and histological risk parameters as defined by cIMPACT-NOW update 8 in meningiomas [10]. Our results extend prior work by directly applying machine learning to these latest molecularly integrated classification criteria and by achieving excellent performance across multiple clinically relevant endpoints.

The ability to non-invasively predict integrated molecular risk (AUC = 0.91) and 1p chromosomal status (AUC = 0.90; 87.5% accuracy) has substantial clinical potential in modern neuro-oncology. According to cIMPACT-NOW update 8, the presence of 1p deletion in combination with 22q deletion and/or NF2 variants warrants assignment of CNS WHO grade 2, even for morphologically grade 1 cases [10]. Accordingly, preoperative 1p prediction facilitates application of these recommendations in scenarios where tissue-based assays are pending or not immediately available, supporting prioritization of confirmatory molecular testing. Preoperative knowledge of molecular risk parameters can directly inform clinical management by enabling individualized patient counseling and shared decision-making, as accurate, non-invasive risk stratification provides early prognostic insight regarding treatment and follow-up [10,13]. It could also guide surgical planning by identification of meningiomas with higher-risk molecular features for consideration of more extensive resection in lesions likely to behave aggressively [3,13]. Irrespective of risk profile, however, gross total resection including the dural attachment remains a principal objective when safely achievable, limiting the extent to which risk prediction should alter standard resection goals. In settings where comprehensive molecular profiling is not feasible for all, radiomics-guided preselection can identify candidates most likely to benefit from additional molecular assays, thereby optimizing resource allocation [10]. From a testing-strategy perspective, a high-sensitivity prescreen is desirable for key alterations (e.g., 1p loss): predicted positives proceed to confirmatory molecular pathology, while predicted negatives (e.g., 1p intact) can be deprioritized, reducing unnecessary testing. Furthermore, integrated radiomic and molecular information may support risk-adapted selection and timing of adjuvant radiotherapy or other interventions in alignment with contemporary EANO and cIMPACT-NOW update 8 recommendations [3,10]. Although the present study does not evaluate recurrence or survival endpoints, a recent systematic review and meta-analysis reported strong performance of radiomics- and machine learning-based models for meningioma recurrence, and prior single-center work using routine preoperative MRI likewise predicted postoperative recurrence and high-grade histology, together supporting the translational potential of imaging-derived risk stratification for longitudinal outcomes [22,23]. However, these outcomes differ from the cIMPACT NOW update 8-aligned molecular endpoints evaluated here. Stratification by predicted molecular risk may facilitate enrollment in studies and clinical trials targeting aggressive or molecularly distinct subtypes, supporting precision-medicine strategies [10]. Nevertheless, tissue-based molecular evidence remains the reference standard for treatment-defining decisions; radiomics should be positioned as complementary decision support.

### 4.2. Biological Basis of Radiomics Findings

The consistent prominence of shape features—particularly tumor flatness and irregularity—across all models, in line with our empirical feature rankings, supports the view that three-dimensional morphology reflects fundamental biological properties of meningiomas [16,24,25]. Consistent with prior observations, irregular growth patterns and reduced sphericity are associated with more aggressive molecular behavior, serving as radiologic surrogates for infiltrative growth—including patterns in which the tumor breaks through the dura mater or extends into venous sinuses, features characteristic of more molecularly aggressive meningiomas [16,24]. The prominence of flatness aligns with recent evidence that sphericity and surface regularity enable non-invasive grading, together indicating loss of shape regularity as a reproducible marker of higher-grade biology [26]. In this context, these geometrically related descriptors—flatness, sphericity, and surface regularity—capture a shared morphologic deviation from a compact, regular shape, which is associated with more infiltrative or irregular growth patterns. Notably, these associations are non-causal; prospective histopathological correlation should further substantiate biological plausibility between morphology and integrated molecular risk.

Additionally, FLAIR-derived GLCM texture features capture microstructural heterogeneity and peritumoral edema, and likely reflect complex interactions between the tumor and its microenvironment. Such edema is not merely passive fluid but may indicate increased vascular permeability, venous congestion, or microscopic tumor cell infiltration into adjacent dura or brain parenchyma [25,27]. Integrating core morphology with FLAIR-based texture therefore profiles both the primary tumor architecture and the surrounding tissue response, yielding a coherent radiogenomic signature and supporting MRI radiomics as a non-invasive surrogate for molecular risk classification.

### 4.3. Comparison with Literature and Performance Validation

Our results strongly align with contemporary evidence on meningioma radiomics: in a systematic review, Patel et al. reported mean AUCs of approximately 0.85–0.89 for grading and biologic feature prediction [12]. The present study extends this literature by directly targeting cIMPACT-NOW update 8 endpoints—integrated molecular risk and chromosome 1p status—thereby increasing clinical applicability relative to WHO-only grading [10].

A persistent limitation, shared both by our work and the field, is the discrimination of WHO grade 2 from grade 3 meningiomas [8]. This challenge reflects not only sample-size constraints but, more fundamentally, a biological continuum at the grade 2/3 boundary—explicitly acknowledged in recent cIMPACT-NOW update 8 on meningioma classification. Grade assignment increasingly depends on specific genomic events, most notably CDKN2A/B homozygous deletion and TERT promoter mutation, rather than histopathology alone [10]. Radiomics derived from conventional MRI sequences does not directly assay these alterations and, accordingly, may only imperfectly reflect this molecular inflection point, reinforcing the continued need for confirmatory tissue-based testing in suspected higher-grade disease.

Importantly, our findings are consistent with prior observations: low-risk biology is most separable, whereas errors cluster at the medium- versus high-risk boundary, consistent with a directed continuum in the imaging phenotype with increasing biological risk [16]. In this context, integrated molecular risk (AUC 0.91) and 1p status (AUC 0.90) were predicted robustly, while WHO grade misclassifications predominantly involved grade-3 cases labeled as grade-2, in line with the molecular overlap highlighted by cIMPACT-NOW update 8 [10]. Accordingly, implementation should pair radiomics with confirmatory molecular testing—and, when indicated, advanced imaging—to ensure accurate risk assignment, particularly at the grade 2/3 boundary.

### 4.4. Technical Advantages and Clinical Implementation

Random forests compute a score for each input feature that reflects how much that feature, on average, reduces misclassification across the many decision trees in the model; these “feature importance” scores make it transparent which MRI-derived variables most influence the final prediction [28]. Our identification of tumor flatness as a key predictor further supports clinical interpretability, with irregular or “en plaque” tumor growth patterns serving as visual indicators of higher molecular risk—findings consistent with previous studies [16,24].

Recent advances in automated tumor segmentation, particularly those driven by the BraTS challenge and toolkit, have enabled robust meningioma delineation with minimal manual correction [29]. A key methodological advantage of this study is the use of a fully automated pipeline that uses BraTS for segmentation and proceeds to feature extraction. This minimizes the considerable inter- and intra-observer variability inherent to manual segmentation, thereby increasing the reproducibility and potential transferability of the models to other clinical centers. These capabilities support end-to-end pipelines, from segmentation to feature extraction and classification—essential for clinical workflow integration. Ensuring adherence to Imaging Biomarker Standardization Initiative (IBSI) recommendations is essential for feature reproducibility and cross-site comparability [21]. Consistent with this rationale, recent multi-center work shows that hybrid radiomic–deep learning models, combined with protocol-aware harmonization, enable reproducible meningioma grading across heterogeneous MRI protocols [30].

### 4.5. Advanced Imaging and Multimodal Integration

While this study used conventional MRI sequences, diffusion-weighted imaging and perfusion MRI merit evaluation to improve risk prediction and tumor grading where standard radiomics is limited [8,24]. Additionally, somatostatin-receptor PET (SSTR-PET) may complement MRI by providing target-specific molecular information that is not directly accessible on routine sequences [25]. Prior work—including our own—indicates that advanced imaging can be particularly helpful for distinguishing molecularly similar yet clinically distinct subtypes, with diffusion and perfusion showing promise at the medium- versus high-risk boundary—a setting in which standard MRI radiomics is often limited [16].

Recent multimodal strategies that combine imaging, genomics, and clinical data have demonstrated superior classification performance and should be prioritized in prospective studies, especially for complex or molecularly ambiguous meningioma subtypes [13].

### 4.6. Limitations and Future Directions

Several limitations warrant acknowledgment. The retrospective design and single-institution, multi-scanner acquisition introduce potential selection and technical biases, despite standardized preprocessing. The limited number of WHO grade 3 cases in the test set (n = 2) restricts definitive conclusions for this subgroup, although this reflects real-world distributions [1]. Class imbalance across endpoints may also have influenced operating characteristics, particularly sensitivity at the medium/high-risk boundary.

The absence of clinical follow-up in this study (e.g., progression-free survival) prevents direct evaluation of prognostic value. While feasibility for MRI-based recurrence prognostication for WHO-grading is supported by recent literature, longitudinal validation is not yet available for the new endpoints evaluated here—integrated molecular risk (low vs. high, cIMPACT-NOW 8) and 1p-deletion status—and will be required to establish prognostic value beyond cross-sectional discrimination. Prospective tracking of outcomes in radiomics-defined high- versus low-risk groups will be crucial to determine whether imaging-based predictions provide additional prognostic information—potentially even beyond molecular pathology in selected situations—while recognizing neuropathological classification as the current reference standard.

Future prospective studies should include: (1) larger cohorts with balanced representation of molecular and histological subtypes; (2) standardized image acquisition together with protocol-aware harmonization across scanners and sites to reduce protocol- and device-related variability; (3) systematic linkage to clinical outcomes; (4) incorporation of advanced imaging where appropriate (diffusion/perfusion MRI and selective SSTR-PET); and (5) external validation across independent sites, vendors, and protocols. Future multi-center external evaluation should use a uniform pre-processing pipeline with protocol-aware feature harmonization to ensure cross-site comparability, consistent with recent multi-center evidence for reproducible grading under heterogeneous MRI protocols [30]. For clinical deployment, models should undergo probability calibration with predefined decision thresholds aligned to clinical use cases, and provide full transparency of feature-extraction parameters and code to ensure reproducibility. These steps are essential to rigorously determine the prognostic value of radiomics-based molecular prediction within integrated molecular–morphologic classification frameworks [16].

## 5. Conclusions

MRI-based radiomics aligned with cIMPACT-NOW update 8 can provide clinically meaningful, non-invasive insights into meningioma biology, with strong performance for integrated molecular risk and 1p status supporting preoperative characterization within clearly defined preoperative use cases. However, the approach is not yet ready for clinical use, and accuracy is lower for current WHO grade classification. Shape-derived morphology, complemented by FLAIR texture, offers interpretable signals consistent with underlying molecular features, while definitive decisions at genomically defined boundaries should continue to rely on tissue-based assays. Prospective, protocolized validation with standardized pipelines and calibrated operating points remains the critical next step to enable careful, reproducible clinical deployment. Longitudinal and multi-center validation will be essential to define prognostic utility and generalizability.

## Figures and Tables

**Table 1:** Radiomics Model Performance for Predicting Molecular Risk, 1p Status, and WHO Grade in Meningiomas.

Endpoint	AUC	Accuracy (%)	Weighted F1-Score	Sensitivity (%)	Specificity (%)
Risk Classification (High/Low)	0.91	91.1	0.91	68.8	100
1p Status (Loss vs. Intact)	0.90	87.5	0.87	62.5	97.5
WHO-Grade (1/2/3)	0.89	76.8	0.75	–	–

**Abbreviations:** AUC, area under the receiver operating characteristic curve; F1-score, harmonic mean of precision and recall. Sensitivity and specificity are listed only for binary outcomes.

## Data Availability

De-identified data supporting the findings are available from the corresponding author upon reasonable request; radiomics feature definitions and the full feature list are provided in Supplementary Table S1.

## Supplementary Materials

The following supporting information can be downloaded at: https://www.mdpi.com/article/10.3390/cancers17223665/s1, Table S1: Radiomics feature definitions and the full feature list.
